# The use of oral benzodiazepines for the management of dental anxiety: a web-based survey of UK dentists

**DOI:** 10.1038/s41415-023-5850-5

**Published:** 2023-05-24

**Authors:** Kathryn Finn, Deborah Moore, Yvonne Dailey, Wendy Thompson

**Affiliations:** 292471104778267299931 Division of Dentistry, University of Manchester, Manchester, United Kingdom; 016126754496286230298 NHS England North West, Liverpool, United Kingdom

## Abstract

**Background** Oral benzodiazepines (OBZs) can facilitate treatment of dentally anxious patients and are widely used in countries such as Australia and the United States. Dentists in the UK prescribe them much less often.

**Aims** To examine OBZ prescribing by dentists in the UK, including patterns of practice, barriers to use, and alternative anxiety management strategies.

**Methods** An online mixed-methods survey was conducted utilising Qualtrics. Participants were recruited via the private Facebook group 'For Dentists, By Dentists' during April to June 2021. Quantitative data were analysed with descriptive statistics and qualitative data with thematic analysis.

**Results** In total, 235 dentists participated, with 91% being general dentists. Half had previously prescribed OBZs, with 36% doing so in the last year. Only 18% were confident in their use. Diazepam was the anxiolytic preferred by respondents. Two-thirds of dentists who had never prescribed anxiolytics were interested in doing so in the future. Concerns about managing anxious patients with OBZs included: inadequate training; confusion about guidelines; medico-legal risk; and issues of general practitioners prescribing anxiolytics to dental patients unbeknown to their dentist.

**Conclusions** A lack of confidence prescribing OBZs for anxiolysis exists among UK dentists. Guidelines should be clarified, and training provided.

**Supplementary Information:**

Zusatzmaterial online: Zu diesem Beitrag sind unter 10.1038/s41415-023-5850-5 für autorisierte Leser zusätzliche Dateien abrufbar.

## Key points

- Most UK dentists are not confident prescribing oral benzodiazepines for the management of anxious patients, even those who have prescribed them in the past. A training need exists for the pharmacological management of anxious patients in general dental practice and clear guidance would be helpful.
- Many dentists had experienced patients attending for treatment who had taken oral benzodiazepines prescribed by their general medical practitioner. Concern was expressed that dentists did not often know when patients had taken these drugs before an appointment and the impact this had on patient safety and consent.
- Diazepam is the drug of choice for most dentists, even though the British National Formulary recommends temazepam when it is important to minimise the effects the following day.

**Supplementary Information:**

Zusatzmaterial online: Zu diesem Beitrag sind unter 10.1038/s41415-023-5850-5 für autorisierte Leser zusätzliche Dateien abrufbar.

## Introduction

Dental anxiety is common, with 12% of adults reporting extreme levels of dental anxiety in the most recent *Adult dental health survey*.^1^ Anxiety has been shown to be a major factor in the avoidance of regular dental care and diversion of patients to medical rather than dental services.^2^^,^^3^ As such, patients with dental anxiety are more likely to have untreated dental disease and to only attend when they have a dental problem.^1^^,^^4^ Furthermore, highly anxious patients may find that their anxiety prevents them from accepting dental interventions, further restricting their access to care.^5^

While non-pharmacological methods, such as behavioural management techniques and cognitive behavioural therapy (CBT), can be successful in managing patient anxiety, a study of dental non-attenders demonstrated that those with high levels of anxiety were less willing to explore these methods.^5^ Pharmacological methods are, therefore, sometimes used to facilitate the delivery of dental care for the most anxious patients. Prescribing of oral benzodiazepines (OBZs) as anxiolytics/hypnotics is widespread but dependence (both physical and psychological) and tolerance occur, particularly if the patient has been taking them regularly for more than a few weeks.^6^ Diazepam and temazepam are the OBZs included in the UK Dental Practitioners Formulary and can be prescribed as pre-medication before clinical procedures.^6^

According to the 2015 Intercollegiate Advisory Committee for Sedation in Dentistry (IACSD) guidelines, conscious sedation can only be provided by dental practitioners who have received additional training.^7^ However, all dental practitioners can prescribe OBZs at anxiolytic doses as pre-medication. Pre-medication is defined as the self-administration of a small dose of an oral sedative to alleviate anxiety, often at home.^7^ Oral sedation is the administration of a much larger dose of an oral sedative at the dental practice.^7^ The distinction between the two relates to the effect on the patient. Sedative doses result in a mild impact on the patient's physiological function and response to verbal stimulus, whereas anxiolytic doses reduce a patient's anxiety while maintaining a normal response to verbal commands and physiological functions are unaffected.^8^ Advice on prescribing OBZs as pre-medication in dental practice is available from the UK Dental Medicines Advisory Service.^8^

A much lower rate of OBZ prescribing by dentists exists in England compared to other countries, such as Australia and the United States.^9^ Even before the COVID-19 pandemic, access to NHS sedation services varied across England.^10^ This study aimed to explore the prescribing of OBZs for anxiety management by dentists in the UK, including prescribing patterns, barriers and enablers to prescribing, and alternative approaches employed to manage dentally anxious patients.

## Methods

An online questionnaire was designed, pilot tested (with 13 general and speciality dentists), and finalised as a combination of demographic, quantitative and qualitative questions (see online Supplementary Information). Participants were recruited through the private Facebook group 'For Dentists, By Dentists' between April and June 2021. The recruitment message explained that the study aimed to explore drug prescribing for patients with dental anxiety. The study sample size was calculated as 138, based on a target of 5% precision around an estimate of the proportion of participants that had ever prescribed OBZ, with 95% confidence. The calculation assumed that 10% of participants would have previously prescribed OBZs (from NHS prescribing data in 2019).^11^ The estimated population size was based on the Facebook group membership of 17,300.

Descriptive statistics were used to present the quantitative results of this study, with confidence intervals (CIs) calculated using the Wilson Score interval within the Qualtrics survey tool and using SPSS software where necessary. Qualitative data from the free-text answers were analysed using thematic analysis.^12^

Ethical approval for the study was granted by University of Manchester UREC (Ref: 2021-11318-18298). All participants consented to participate in the study and to have their data used as part of the research.

## Results

In total, 235 eligible dentists participated, of which half (120/235 = 51.1%; 95% CI [44.7-57.4]) had prescribed OBZs to patients for anxiolysis. Drop out through the survey was 11% (n = 26), meaning later questions in the survey were answered by fewer dentists. The results presented below indicate the total number of respondents who answered the related question.

### Demographics

Most respondents were women (139/235 = 59.1%), aged 22-40 years (142/235 = 60%), general dental practitioners (213/235 = 90.6%) and had qualified in the UK (214/235 = 91.1%). Little difference existed in OBZ prescribing experience between general and speciality dentists (51.2% vs 50.0%) (see Table 1). Those qualified before 1992 were twice as likely to have prescribed OBZs as those qualified since 2012 (>80% compared with 37%).

**Table 1:** Demographics of survey respondents

Demographic		Total number of respondents (n = 235)	Proportion who had prescribed OBZs (95% CI)
Sex	Male	92	58.7% (48.5-68.2)
	Female	139	46.8% (38.7-55)
	Prefer not to say	4	*
Type of dentist	General dentist	213	51.2% (44.5-57.8)
	Speciality dentist/trainee	22	50.0% (30.7-69.3)
Country qualified	UK	214	52.8% (46.1-59.4)
	Non-UK	21	33.3% (17.2-54.6)
Year qualified	1972-1981	3	*
	1982-1991	25	80% (60.9-91.1)
	1992-2001	33	69.7% (52.7-82.6)
	2002-2011	76	51.3% (40.3-62.2)
	2012-2021	98	36.7% (27.9-46.6)
Key: * = Numbers too small for statistical analysis			

### Patterns of OBZ prescribing

Of 120 dentists who had prescribed OBZs, most prescribed as pre-medication (103/120 = 85.8%; 95% CI: 78.3-91.5); 16 for temporomandibular joint problems and eight solely for conscious sedation. One-third (n = 41) had done so most recently in the last year, while one-quarter (n = 29) reported that it was over five years ago. Two-thirds of the dentists who did not currently prescribe them for anxiolysis (108/161 = 67%) would be interested in doing so in the future.

One-third of respondents (85/229 = 37.1%) reported having asked a general medical practitioner (GP) to prescribe OBZs as anxiolysis for a patient.

**Fig. 1:**
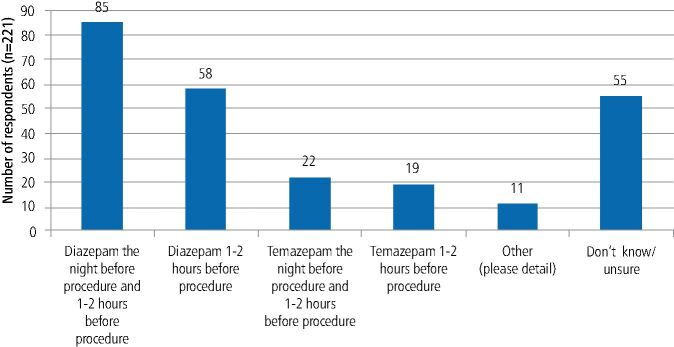
Preferred OBZ prescribing regimen for a fit and well anxious adult to facilitate the surgical removal of a tooth (participants could select more than one option, therefore the total number of responses is greater than the number of respondents)

In response to a scenario about OBZ prescribing for anxiolysis, most prescribed diazepam the night before a procedure and/or two hours before a procedure (67.7% = 143/211) (see Figure 1).

### Barriers and enablers to OBZ prescribing

**Fig. 2:**
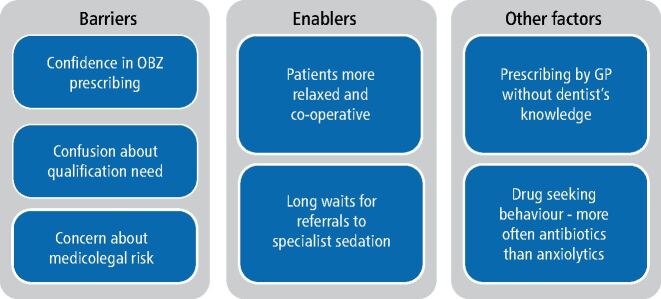
Summary of barriers, enablers and other complicating factors

Barriers, enablers and other factors influencing OBZ prescribing are detailed below and summarised in Figure 2.

#### Barrier: confidence in OBZ prescribing

Just 17.8% of all respondents (n = 39/219) reported high or very high confidence in OBZ prescribing. More than 70% (n = 155/219) wanted further training and some highlighted the lack of clear guidance on OBZs as pre-medication:

- 'I'm not sure about guidance with prescribing oral medication for dental anxiety and so have avoided it for a number of years'.

#### Barrier: confusion about qualification requirements

Dentists who had never prescribed OBZs cited not having a formal sedation qualification (n = 54), medico-legal risk (n = 43), a preference for other anxiety management approaches (n = 28), concerns about safety (n = 8), inadequate remuneration (n = 6) and concerns about drug effectiveness (n = 2) as barriers to their prescribing.

#### Barrier: concern about medico-legal risk

Safety concerns included issues of access to a patient's complete medical history, which may be important when prescribing OBZs, and the risk of contributing to substance misuse disorder:

- 'Unsure of patient's exact medical histories. It's easier in secondary care to readily access GP records than it is as a general dental practitioner so easier to be more confident there will be no drug interactions'.

By working with the patient's GP, dentists felt more comfortable as they had access to a complete medical history, which is important for identifying potential drug interactions and any concerns about substance misuse disorder:

- '[OBZs are] a controlled drug and drug of abuse - I am concerned my patients may try and coerce myself/others into prescribing oral sedatives more regularly if I make it common practice'.

Perceived difficulties also related to ensuring that the dose given would not inadvertently cause the patient to be sedated:

- 'One patient was very drowsy and had to stay in the spare surgery to recover - despite our practice not being set up as a sedation practice'.

#### Enabler: patients more relaxed and cooperative

Many dentists advocated the benefits of treating patients who had taken OBZs as oral pre-medication:

- 'I believe it improved the ability to give care because the patient was less anxious and more cooperative'
- 'It made the procedure easier as the patient seemed a lot more relaxed'.

#### Enabler: long waits for sedation services

Poor access to NHS services for anxious patients were reported as an incentive to using OBZs to facilitate care in general dental practice:

- 'Long waits for sedation on NHS, so think it's worth prescribing and trying the oral benzo [sic], as delayed treatment can lead to loss of a tooth which may otherwise have been saved'.

#### Other factors: prescribing by GPs

Nearly half of dentists (100/221 = 45.2%) reported having treated patients who had taken oral sedatives prescribed by a GP without their input (and sometimes even without their knowledge):

- 'The problem comes when the GP has prescribed and [the patient] has taken the medication without my knowledge. One patient I believed was drunk'
- 'Makes it difficult and challenging: the consent process is compromised. Patient safety was compromised as did not always have an escort'.

#### Other factors: drug-seeking behaviour

Anxiolytics were not commonly requested (25/210 = 11.7%). Respondents identified antibiotics as the most likely drugs to be requested by anxious dental patients (153/210 = 71.5%), followed by opioid analgesics, such as dihydrocodeine (46/210 = 21.5%) and non-opioid analgesics, such as ibuprofen (38/210 = 17.8%).

### Alternative approaches to anxiety management

Many dentists reported avoiding the use of OBZs, with most preferring behavioural management techniques (see Table 2).

**Table 2:** Proportion of dentists using each of the other anxiety management techniques

Choice	Proportion	Confidence interval (95%)
Behavioural management techniques	89.5%	84.7-92.9%
Intravenous sedation	31.1%	25.3-37.5%
Systematic desensitisation	22.4%	17.4-28.3%
Inhalation sedation	16.9%	12.5-22.4%
Cognitive behavioural therapy	9.6%	6.4-14.2%
Hypnosis	7.3%	4.5-11.5%
Other	7.3%	4.5-11.5%
None	5.0%	2.8-8.8%
Acupuncture	3.2%	1.6-6.4%

## Discussion

A general lack of confidence about OBZ prescribing exists, together with a desire for further training in prescribing anxiolytics, even among current prescribers. More recently qualified dentists were less likely to have ever prescribed OBZs. Treating patients who had taken anxiolytics was generally reported to be a positive experience, with care facilitated by more relaxed and cooperative patients. Most dentists preferred, however, to use behavioural management techniques to facilitate treatment of anxious patients. Long waiting lists for referral to specialist NHS services for anxious patients was motivating some dentists to consider prescribing anxiolytic premedication to their patients in general dental practice. However, changes in the legal framework for controlled drugs and the introduction of the IACSD sedation guidelines in 2015 introduced confusion for many, including whether additional qualifications are required for prescribing anxiolytic pre-medication.^7^

Diazepam was the preferred OBZ among respondents, consistent with routinely collected NHS prescribing data.^9^ However, it is more prone to interactions and has a longer half-life than temazepam.^6^ For these reasons, the British National Formulary recommends temazepam as more suitable when it is important to minimise any residual effect the following day.^6^ By contrast, the Scottish Dental Clinical Effectiveness Programme (SDCEP) drug prescribing guidelines recommend only diazepam as pre-medication.^13^ These sorts of discrepancies between the two documents are examples of the lack of clear guidance for dentists on OBZ prescribing.

However, the UK Misuse of Drugs Regulations 2001 classifies diazepam as a Schedule 4 controlled drug (CD) and temazepam as a Schedule 3 CD (which has additional requirements in relation to prescribing).^14^ For NHS patients, the standard FP10D prescription form can be used for both diazepam and temazepam. For private patients, Schedule 3 drugs must be prescribed on a private CD prescription form (FP10PCD) which can be obtained from the NHS, even if the dentist has no contractual relationship with the NHS.^8^ Various additional legal requirements for prescribing Schedule 3, but not Schedule 4, CDs exist, including the requirement to specify 'for dental treatment only'.^8^

Confusion was also expressed in the study about whether dentists without additional qualifications can prescribe OBZs. While the IACSD's *Standards for conscious sedation in the provision of dental care* and the SDCEP's *Conscious sedation in dentistry* guidelines both cover pre-medications, some ambiguity is clear.^6^^,^^13^ These inconsistencies and ambiguities within current guidance could be a contributing factor in dentists' self-reported lack of confidence in prescribing OBZs. USA guidelines are much more explicit on the requirements that a dentist must satisfy before prescribing OBZs at anxiolytic doses.^15^ Further research is indicated to produce clear UK guidance about pre-medication, including doses and the need for additional qualifications.

This study demonstrates that some GPs have been playing a role in the management of dentally anxious patients by prescribing OBZs, both with and without the involvement of their patient's dentist. No previous research has specifically explored the prescription of OBZs by GPs for dental reasons; however, previous studies show that GPs are often approached by patients for the management of dental conditions and that dental anxiety and difficulty accessing dental services are contributing factors.^3^^,^^16^^,^^17^ This places an increasing burden on GPs, as well as posing a significant medico-legal risk for dentists treating patients without knowledge that they had taken OBZs.^18^^,^^19^ Displacement of anxious dental patients to GPs may help explain the significantly lower OBZ prescription rate found in England compared to the USA and Australia.^9^ GPs have been advised by the British Medical Association (and required by NHS commissioners in many areas) that they should not be managing dental conditions (including prescribing).^19^^,^^20^ A further patient safety concern relating to the prescribing of OBZs was the lack of access for dentists to a patient's complete medical history. Summary Care Records are an electronic record of important patient information, created from GP medical records.^21^ They can be seen and used by authorised staff in other areas of the health and care system involved in the patient's direct care, such as community pharmacists.^21^ Extending access to primary care dentists would improve dental patient safety generally and could facilitate the safe prescription of OBZs by dentists. Further research is needed to develop strategies to safely manage dentally anxious patients across primary care settings, including understanding patient perspectives on OBZs.

The main strength of this study was that it provided insight into the previously underexplored area of OBZ prescribing by UK dentists. The use of social media for recruitment allowed the survey to be conducted on a national scale. However, compared to the demographics of UK dentists registered with the General Dental Council, this recruitment strategy has resulted in recruitment bias towards a cohort of dentists who were more likely to have been trained in the UK (91% of respondents vs 74% of GDC-registered dentists) and slightly younger (60% of respondents were 22-40 years old vs 48% of GDC-registered dentists).^22^ Selection bias also seems to have been an issue, with those trained to provide conscious sedation more likely to participate, as evidenced by the considerable number of dentists indicating that they had used intravenous sedation to manage dental anxiety. The proportion of respondents with experience of prescribing OBZs was higher than originally estimated in the sample size calculation, which had the effect of making the 95% CIs around this estimate wider than originally intended. However, as this is a hypothesis-generating, exploratory study, the intention was to gather initial information rather than produce a very accurate and/or representative estimate. Given the relatively large proportion of participants who had previously prescribed OBZs, and the likelihood of the survey respondents self-selecting due to an interest in the topic of OBZ use, the finding of low knowledge and confidence is particularly interesting and suggests that there may be a significant training need nationally. That younger dentists were less likely to have prescribed OBZs suggests that dental schools may need to boost their teaching about the pharmacological management of anxious dental patients with OBZs as pre-medication.

The authors are not recommending wide-spread use of OBZs for the management of dental anxiety, as it is well-known that these drugs have the potential for abuse and are associated with side effects.^23^ While sedation can be an effective adjunct for anxiety management, other techniques, such as behaviour management techniques, are preferred by many respondents, and CBT has been shown to aid long-term reduction of anxiety.^24^^,^^25^^,^^26^^,^^27^ The development of pathways to care for anxious dental patients, involving both pharmacological and non-pharmacological elements, should improve access to oral health care for dentally anxious patients and reduce the burden from dental patients on GPs. Further research to develop clear national guidelines on safe OBZ prescribing can support implementation of these care pathways.

## Conclusion

A lack of confidence in prescribing OBZs for anxiolysis exists among UK dentists. Diazepam was preferred by dentists despite its residual effects lasting longer when compared to temazepam. GPs are prescribing OBZs both with and without dentists' input to alleviate patients' dental anxiety. This impacts on both patient safety and ability to provide valid consent during dental appointments. In view of these findings, guidelines for dentists and GPs should be clarified and training provided to enhance patient safety, reduce the burden on GPs, and broaden access to dental care for anxious patients.

## Supplementary Information

Supplementary Information (PDF 154KB)
