# Supplementary material for: The use of oral benzodiazepines for the management of dental anxiety: a web-based survey of UK dentists
Source: Br Dent J. 2023 May 24:1–5. Online ahead of print. doi: 10.1038/s41415-023-5850-5 (PMC10208682; doi:10.1038/s41415-023-5850-5)
Supplement: Supplementary file 1 — Supplementary Information (PDF 154KB) [file 41415_2023_5850_MOESM1_ESM.pdf]

The Use of Oral Benzodiazepines for the Management of Dental Anxiety by UK Dentists

---

Start of Block: Oral Benzodiazepine use for the management of dental anxiety

Q1 Before agreeing to participate with this study please read the participant information sheet.

You can find out more information about how the University handles your data in the privacy policy.

◯ I have read the participant information and consent to take part in this study

◯ I do not consent to take part in this study

Skip To: End of Survey If Before agreeing to participate with this study please read the participant information sheet. ... = I do not consent to take part in this study

Q2 Are you a registered dentist who has practised clinical dentistry in the UK within the last 2 years?

◯ Yes

◯ No

Skip To: End of Survey If Are you a registered dentist who has practised clinical dentistry in the UK within the last 2 years? = No

Q3 Do you identify as:

- ◯ Male
- ◯ Female
- ◯ In another way
- ◯ Prefer not to say

Q4 Your age group

▼ 22-30 ... Prefer not to say

JS

Q5 When did you qualify as a dentist?

Year	▼ 1900 ... 2049

Q6 Where did you gain your primary dental qualification?

- ◯ UK dental school
- ◯ EU dental school
- ◯ Non-EU dental school

Q7 Which country or countries do you currently practise dentistry?

- [ ] England
- [ ] Scotland
- [ ] Wales
- [ ] Northern Ireland
- [ ] Other (please state)

____

Q8 Which sector(s) do you currently work in? (select all that apply)

- [ ] General Dental Practice - NHS
- [ ] General Dental Practice- Mixed
- [ ] General Dental Practice - Private
- [ ] Community Dental Service
- [ ] Secondary Care (dental hospital)
- [ ] Secondary Care (general hospital)
- [ ] University
- [ ] Other (please state)

____

Q9 Are you a registered specialist or undertaking specialty training?

- ○ No
- ○ Yes- Specialist
- ○ Yes- Specialty trainee

Skip To: Q11 If Are you a registered specialist or undertaking specialty training? = No

Q10 Please indicate which specialty or specialties you are registered or completing specialty training in (select all that apply).

	Specialist	Specialty trainee
Endodontics	☐	☐
Restorative Dentistry	☐	☐
Oral Medicine	☐	☐
Oral Surgery	☐	☐
Orthodontics	☐	☐
Paediatric Dentistry	☐	☐
Periodontics	☐	☐
Prosthodontics	☐	☐
Special Care Dentistry	☐	☐

Q11 Have you ever prescribed oral benzodiazepines (temazepam or diazepam) to your adult patients for the purpose of anxiety management (as oral pre-medication or conscious sedation)?

○ Yes

○ No

Skip To: Q16 If Have you ever prescribed oral benzodiazepines (temazepam or diazepam) to your adult patients for... = No

---

Q12 How do you use oral benzodiazepines (temazepam or diazepam) for anxiety management? (select all that apply)

☐ Oral pre-medication

☐ Conscious sedation

☐ Not sure

Q13 In the past 12 months, when patients have been unable to cope with treatment under local anaesthetic alone, for how many of these patients have you prescribed oral benzodiazepines?

○ None of my patients

○ Some of my patients

○ Most of my patients

○ All of my patients

○ Not sure

Q14 When was the last time you prescribed oral benzodiazepines for the purpose of anxiolysis?

- ◯ Less than 1 year ago
- ◯ 1-5 years ago
- ◯ Over 5 years ago
- ◯ Not appicable - Never prescribed

Display This Question:

If Have you ever prescribed oral benzodiazepines (temazepam or diazepam) to your adult patients for... = Yes

Q15 What consenting procedure do you use when prescribing oral benzodiazepines for anxiety management?

- ◯ Written Consent (consent form)
- ◯ Verbal Consent
- ◯ Implied Consent
- ◯ Other (please state) ____

Display This Question:

If Have you ever prescribed oral benzodiazepines (temazepam or diazepam) to your adult patients for... = No

Q16 Why have you never prescribed oral benzodiazepines (either as an oral pre-med or conscious sedation) for managing anxious patients? (Select all that apply)

- ☐ Do not believe they are effective
- ☐ Feel they are unsafe to use
- ☐ Inadequate training
- ☐ Lack of confidence
- ☐ Medicolegal risk
- ☐ Not adequately remunerated
- ☐ No formal sedation qualification
- ☐ Prefer other anxiety management approaches
- ☐ Other (please comment) ____________________

Q17 Have you ever asked a patient's GMP to prescribe oral benzodiazepines for the purpose of anxiolysis for a dental procedure?

- ○ Yes
- ○ Not sure
- ○ No

Q18 Have you ever prescribed oral benzodiazepines to dental patients for any reason other than anxiolysis e.g. TMD?

○ Yes

○ No

Display This Question:

If Have you ever prescribed oral benzodiazepines to dental patients for any reason other than anxiol... = Yes

Q19 For which clinical indication(s) (other than anxiolysis) did you prescribe oral benzodiazepines?

____

Q20 Which of the following prescribing regimen would you consider for a fit and well (ASA 1) anxious adult, under 65 years old, requiring an anxiolytic dose of oral benzodiazepines to facilitate the surgical removal of a tooth? (Select all that apply)

▢ Diazepam 1-2 hours before procedure

▢ Diazepam the night before procedure and 1-2 hours before procedure

▢ Temazepam 1-2 hours before procedure

▢ Temazepam the night before procedure and 1-2 hours before procedure

▢ Don't Know/Unsure

▢ Other (please detail) ____

Q21 Do you have experience of treating patients who have been prescribed oral sedatives for their dental treatment by another health care professionals e.g. GMP, without your input?

- Yes
- No
- Maybe

Display This Question:

If Do you have experience of treating patients who have been prescribed oral sedatives for their den... = Yes

Q22 How did this impact on your care of the patient(s)?

____

____

____

____

____

Q23 What other techniques do you regularly use to manage adult patients' dental anxiety? (tick all that apply)

- ☐ Acupuncture
- ☐ Behavioural management (e.g. positive reinforcement, distraction, stop-start signals, 'tell, show, do' sequence, modelling)
- ☐ Cognitive behavioural therapy
- ☐ Hypnosis
- ☐ Inhalation Sedation
- ☐ Intravenous Sedation
- ☐ Systematic Desensitisation
- ☐ None
- ☐ Other (please detail)

____

Q24 If you don't currently prescribe oral benzodiazepines for anxiolytic purposes would you be interested in doing so in the future?

- ○ Yes
- ○ No
- ○ Not Sure
- ○ N/A - already prescribing

Q25 How would you rate your confidence in prescribing oral benzodiazepines to adult patients for the purpose of anxiolysis?

- ◯ Very high
- ◯ High
- ◯ Moderate
- ◯ Low
- ◯ Very low

Q26 Do you feel that you require any further training on the prescription of oral benzodiazepines?

- ◯ Yes
- ◯ No
- ◯ Not Sure

Q27 What issues or concerns do you have around care provision for dentally anxious patients?

____

____

____

____

____

Q28 Do you notice that anxious patients are more likely to prefer/request a particular type of dental care? (Select all that apply)

- ☐ Advice only
- ☐ Analgesics - non-opioid (e.g. paracetamol, ibuprofen)
- ☐ Analgesics - opioid (e.g. codeine based drugs)
- ☐ Antibiotics
- ☐ Anxiolytics
- ☐ Dental procedures
- ☐ Not sure
- ☐ No
- ☐ Other ____

End of Block: Oral Benzodiazepine use for the management of dental anxiety

---
